# Penetration depth and nonlocal manipulation of quantum spin hall edge states in chiral honeycomb nanoribbons

**DOI:** 10.1038/s41598-017-07994-z

**Published:** 2017-08-08

**Authors:** Yong Xu, Salah Uddin, Jun Wang, Jiansheng Wu, Jun-Feng Liu

**Affiliations:** 1 Department of Physics, South University of Science and Technology of China, Shenzhen, 518055 China; 2 Department of Physics, Southeast University, Nanjing, 210096 China

## Abstract

We have studied numerically the penetration depth of quantum spin hall edge states in chiral honeycomb nanoribbons based on the Green’s function method. The changing of edge orientation from armchair to zigzag direction decreases the penetration depth drastically. The penetration depth is used to estimate the gap opened for the finite-size effect. Beside this, we also proposed a nonlocal transistor based on the zigzag-like chiral ribbons in which the current is carried at one edge and the manipulation is by the edge magnetization at the other edge. The difficulty that the edge magnetization is unstable in the presence of a ballistic current can be removed by this nonlocal manipulation.

## Introduction

Two-dimensional topological insulators with honeycomb structure are promising platforms to develop topological electronic devices. Such honeycomb materials with considerable spin-orbit coupling include group IV two dimensional crystals (graphene^1^, silicene^2–4^, germanene^5, 6^, and stanene^7–9^), the double layer perovskyte iridates^10, 11^ and metal organic frameworks (MOF)^12^. The honeycomb monolayer of Bi has also attracted recent attention for realizing large-gap quantum anomalous Hall insulator^13^. Experimentally, silicene has been synthesized on different substrates^2, 3, 14, 15^ and silicene-based field-effect transistors have been recently fabricated at room temperature^16^. In the devices based on such materials, the transport of electrons via quantum spin hall (QSH) edge states is immune to the time-reversal symmetric disorder and defects. The penetration depth of these edge channels is crucial to the device design based on a nanoribbon with finite width because the fine-size effect opens a gap in the edge channels^17^. Different from the situation in the Bernevig-Hughes-Zhang model^18–21^, the penetration depth of these edge states depends on the edge orientation in honeycomb materials. For two high-symmetry directions, it is antiproportional to the spin-orbit gap for the armchair edge, but remains shorter than the lattice constant for the zigzag edge^22, 23^. For low-symmetry directions, the Fermi velocity of edge states in general chiral ribbons^24–29^ is shown to be dependent on the edge orientation^30^. But the penetration depth, which is more important to device applications based on nanoribbons with small sizes, still remains unclear.

On the other hand, the manipulation of topological device is also challenging. Since there exists spontaneous magnetization in the zigzag edge, it seems that the manipulation of the edge current by the edge magnetization in zigzag ribbon based device is promising. However, the ground state with the spontaneous edge magnetization becomes unstable in the presence of a ballistic current through the ribbon^31^. This difficulty can be overcome by nonlocal manipulation^32^ of the edge current. In this nonlocal manipulation, the current is always carried only at one edge, while the operational edge magnetization lies at the other edge. The manipulation is in virtue of the interference effect between the edge states at two edges, namely, the so-called finite-size effect. To achieve such a nonlocal manipulation, two conditions should be satisfied: one is the presence of stable edge magnetization, the other is the considerable penetration depth of the edge states. Although zigzag ribbons host a large magnetization at the current-free edge, the penetration depth is too small to cause the coupling between the edge states at two edges. In this context, the zigzag-like chiral ribbon is the only choice to realize a nonlocal manipulation via the edge magnetization.

In this work, we investigate numerically the penetration depth of quantum spin hall edge states in chiral honeycomb nanoribbons based on the Green’s function method. We show the evolution of the penetration depth during the change of the edge from zigzag to armchair direction. The penetration depth for armchair-like chiral ribbons is much larger than that from zigzag-like ones. For the purpose of achieving a nonlocal manipulation of the edge current by use of the edge magnetization, the intermediate chiral ribbon between the two classes is the best choice because of the coexistence of the edge magnetization and considerable penetration depth.

The rest of the paper is organized as follows. In section II we present the model and formalize the method to calculate the penetration depth based on the Green’s function technique. In section III, we present the results of penetration depths for various chiral edges and use them to fit the finite-size induced gaps opened in the edge channels. In section IV, we propose the nonlocal transistor based on the zigzag-like chiral ribbons. Finally, a brief summary is given in section V.

## Model and Methods

We start from the tight-binding Hamiltonian of honeycomb lattice with intrinsic spin-orbit coupling (SOC)^1^

1 $$H=-t\sum _{\langle ij\rangle \alpha }{c}_{i\alpha }^{\dagger }{c}_{j\alpha }+i\frac{\lambda }{3\sqrt{3}}\sum _{\langle \langle ij\rangle \rangle \alpha \beta }{\nu }_{ij}{\sigma }_{\alpha \beta }^{z}{c}_{i\alpha }^{\dagger }{c}_{j\beta },$$

where ${c}_{i\alpha }^{\dagger }$ and ${c}_{i\alpha }$ are, respectively, the creation and annihilation operators of an electron on site *i* with spin *α* and 〈*ij*〉/〈〈*ij*〉〉 run over all the nearest/next-nearest-neighbor hopping sites. The first term represents the usual honeycomb lattice Hamiltonian in the nearest-hopping approximation, while the second term represents the intrinsic SOC where *σ* = (*σ*_*x*_,*σ*_*y*_,*σ*_*z*_) are the Pauli matrices of spin and *ν*_*ij*_ = + 1(−1) if the next-nearest-neighbor hopping is anticlockwise (clockwise) with respect to the positive *z* axis. The bulk state of the system is the well-known QSH insulator, or the two-dimensional topological insulator with a gap of 2*λ*.

**Figure 1:**
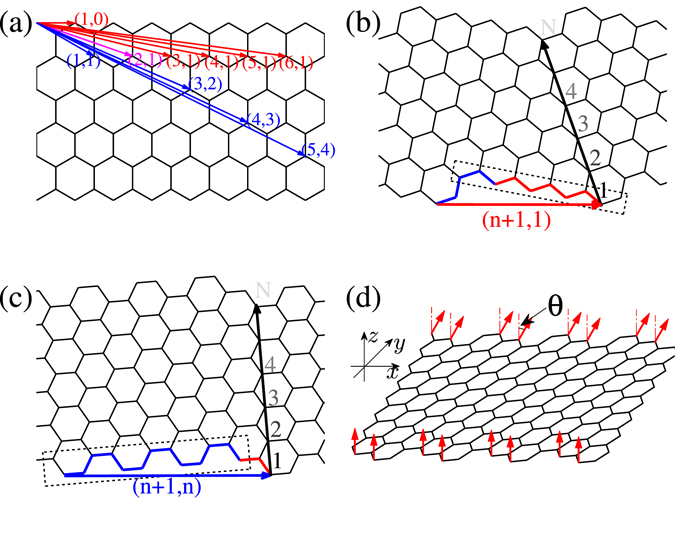
(**a**) The chiral orientations and corresponding indices (*n*,*m*). (**b**) Zigzag-like chiral ribbons with indices (*n* + 1,1). (**c**) Armchair-like chiral ribbons with indices (*n* + 1,*n*). (**d**) Nonlocal transistor controlled by the edge magnetization based on chiral ribbon (2,1).

Next we investigate the penetration depth of QSH edge states. For this purpose, we consider a semi-infinite honeycomb lattice with an edge along the direction labelled by the index (*n*,*m*) (see Fig. 1(a)). Here, the edge of (*n*,*m*) represents a repeating structure composed of *n* − *m* zigzag units and *m* armchair units^33^. The correspondence between the chirality angle and the notation (*n*,*m*) is shown in ref. 33. With these notations, we can denote the gradual transition from zigzag direction to armchair direction. We refer to (1,0), …, (*n* + 1,1), …, (4,1), (3,1) as zigzag-like chiral directions and (3,2), (4,3), …, (*n* + 1,*n*), …, (1,1) as armchair-like chiral directions with *n* > 3. The (2,1) direction is the intermediate direction between two classes.

To find the penetration depths of edge states, we calculate the surface Green’s function for a few layers at the edges. The surface Green’s function can be numerically evaluated by the recursive method for a fixed energy and longitudinal momentum as

$${G}^{S}(E,{k}_{x})=\frac{1}{E-{H}_{D}-{V}^{\dagger }{G}^{S}(E,{k}_{x})V},$$

where *H*_*D*_ is the Hamiltonian of the edge region of interest containing a few layers, *V* is the hopping matrix between two successive regions. The particle density for layer *l* counting from the edge can be obtained by ${\rho }_{l}=-\frac{1}{\pi }Im[Tr\{{G}_{ll}^{S}\}]\mathrm{.}$ Then we fit the decay of the particle density from the edge with the exponentially decaying pattern $\rho (y)\propto \exp \,(-2y/\xi ),$ where *y* is the distance from the edge and *ξ* is the penetration depth of the wave function of the edge state.

### Penetration depth and finite-size induced gap

**Figure 2:**
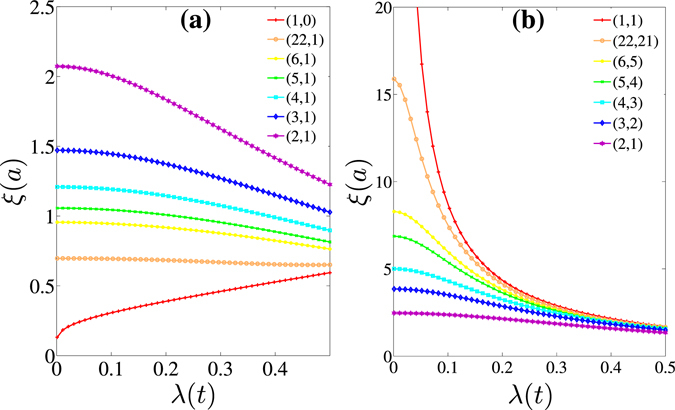
Penetration depths of QSH edge states as functions of the strength of SOC for (**a**) zigzag-like chiral edges with indices (*n* + 1,1) and (**b**) armchair-like chiral edges with indices (*n* + 1,*n*). The results of perfect zigzag edge (1,0) and armchair edge (1,1) are also included as a reference. The Fermi energy is set to *E*_*F*_ = 0.

Figure 2 shows the penetration depths of edge states as functions of the strength of SOC for the chiral edges with various orientations. The Fermi energy is set to *E*_*F*_ = 0. At *E*_*F*_ = 0, the edge state is the most localized. With increasing |*E*_*F*_|, the Fermi energy becomes nearer to the bulk band, and the penetration depth increases. For the class of zigzag-like edges, the penetration depths are less than 2.1*a* with *a* being the lattice constant, and much smaller than that for armchair-like edges. Different from the linearly increasing behavior of *ξ* with increasing *λ* for the perfect zigzag edge (which is consistent with the result $\xi \simeq a\lambda /t$ in Ref. 22), the decay depth decreases with increasing *λ* for the other zigzag-like edges. The decreasing of decay depth with increasing *λ* become slower as the orientation approaches to the perfect zigzag direction, but the linearly increasing behavior for the perfect zigzag edge is hard to recover from (*n* + 1,1) zigzag-like edges even if *n* is very large. Our further numerical calculations show that this situation is the same for the zigzag edge with very few edge impurities or defects. It implies that the linearly increasing behavior for the perfect zigzag ribbon is very frangible. We think the main difference between perfect zigzag edge (1,0) and zigzag-like (*n* + 1,1) chiral edges may be as follows. For the perfect zigzag edge, the edge state is completely localized at one sublattice (*A* sites or *B* sites). For (*n* + 1,1) edges even with large *n* or the zigzag edge with few impurities, the edge state is localized at both *A* sites and *B* sites.

For the class of armchair-like edges, the penetration depths are much larger. From the inverse proportional decreasing feature of the perfect armchair edge (which is consistent to the result $\xi =\sqrt{3at}/(2\lambda )$ in ref. 22), the decreasing of *ξ* as a function of *λ* is getting slower when the armchair-like edge is approaching to the intermediate edge (2,1). It is shown in Fig. 2(b) that especially for a given small *λ*, the penetration depth decreases quickly with increasing *n* for the armchair-like edges labelled by (*n* + 1,*n*).

**Figure 3:**
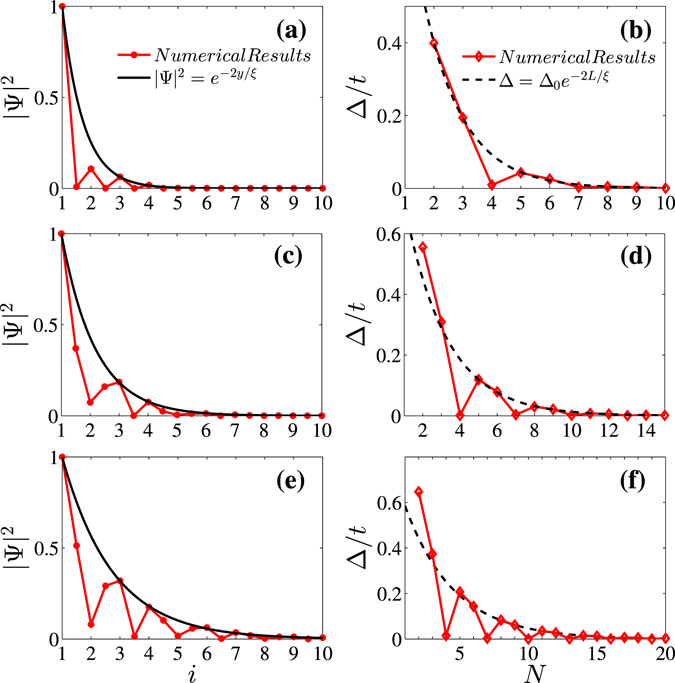
The exact numerical results of the decay of the particle density (left panels) for QSH edge states and the gap of the chiral ribbon with finite width *N* (right panels) are fitted by the formula,respectively. (**a**) and (**b**) are for the ribbon (3,1), (**c**) and (**d**) are for the ribbon (2,1), and (**e**) and (**f**) are for the ribbon (3,2). The strength of SOC is set to *λ* = 0.1*t*. The Fermi energy is set to *E*_*F*_ = 0.

Figure 3(a,c) and (e) show the fitting of numerically determined decaying density of the particle with the exponential law for QSH edge states at three chiral edges (3,1), (2,1), and (3,2) respectively. The penetration depth determined by the fitting can also be used to predict the gap opened in a QSH edge channel due to the finite-size effect in the nanoribbon geometry. The gap is induced by the overlap of two edge states in the ribbon and can be related to the penetration depth by ref. 22 ${\rm{\Delta }}={{\rm{\Delta }}}_{0}\exp (-2L/\xi )$ where *L* is the width of the physical width of the ribbon. The exact gap can be numerically determined by the direct diagonalization of the tight-binding Hamiltonian expressed in Eq. (1) in the ribbon geometry. Figure 3(b,d) and (f) show the fitting of the exact gap as a function of the ribbon width with the penetration depth for three chiral edges (3,1), (2,1) and (3,2) respectively. The good fitting implies that the relation between the gap and the penetration depth derived in the case of perfect armchair ribbon in ref. 22 is also applicable to the chiral ribbons.

### Nonlocal manipulation of QSH edge states

**Figure 4:**
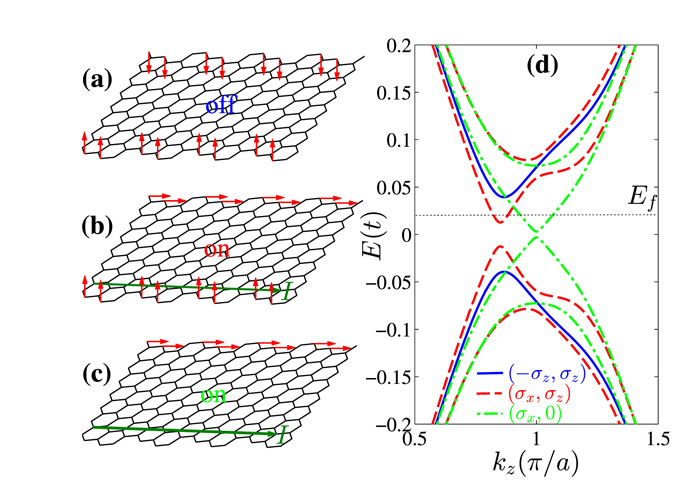
(**a**–**c**) Schematic diagrams of the nonlocal transistor manipulated by the magnetization orientation at the upper edge while the current flows at the lower edge. (**a**) Is for the off-state, (**b**) and (**c**) are for the two on-states. (**d**) Spectrum for the QSH edge channels for the nonlocal transistor based on a chiral ribbon (2,1) with width *N* = 8. The dotted line is the position of the Fermi energy. Three kinds of magnetization configurations shown in (**a**–**c**) are considered. Other parameters are *λ* = *h* = 0.1*t*.

As discussed previously, the zigzag-like chiral ribbon is the good choice to realize a nonlocal manipulation of the QSH edge current due to the coexistence of edge magnetization and considerable penetration depth. The intermediate chiral ribbon (2,1) is the best choice for its biggest penetration depth and maintaining of edge magnetization^33^. A nonlocal transistor controlled by the edge magnetization based on the chiral ribbon (2,1) is illustrated in Fig. 1(d) and Fig. 4(a–c). The current flows along the lower edge and is controlled by the orientation of the edge magnetization at the upper edge. The magnetization is considered only at the outermost sites of zigzag edge chains (shown in Fig. 1(d) and Fig. 4(a–c) with red arrows) as a spin-split on-site energy

2 $${H}_{M}=h\sum _{i}{({\sigma }_{z}\cos \theta +{\sigma }_{x}\sin \theta )}_{\alpha \beta }{c}_{i\alpha }^{\dagger }{c}_{i\beta },$$

where *h* is the strength of the edge magnetization and *θ* is the angle between the magnetizaiton orientation and the *z*-axis.

Although the spontaneous magnetic order at the QSH edge tends to open a gap and leads to an insulating phase^34^, it is possible to employ an external ferromagnet to control the magnetization orientation at each edge. In the operation of the nonlocal transistor, the magnetization at the lower edge is fixed to the *z*-axis. In the off-state, the magnetization at the upper edge points to the −*z* direction. The QSH edge states at two edges are coupled due to the finite-size effect and lead to a gap shown in the solid line in Fig. 4(d). Note that the edge channels are two-fold degenerate in the spin space. The current is blocked when a bias is applied, because the Fermi energy lies in the gap. When the upper magnetization is modulated to the *x* direction, the edge channel at the upper edge opens a big gap and thus does not interfere with the lower edge channel any more. The dashed lines in Fig. 4(d) show that the gap in the lower channel becomes much smaller. The remaining small gap is due to the upper magnetization along the *x* direction which provides a small probability of back-scattering in the lower channel. The Fermi energy lies above this small gap and thus the transistor is in the on-state. If the current carried by the lower channel is big enough, the lower magnetization becomes unstable and vanishes finally. The dash-dotted curves in Fig. 4(d) show that the gap becomes very small when the lower magnetization is absent. It means that the on-state can be kept to support a large edge current.

## Conclusion

In conclusion, we numerically investigate the penetration depth of QSH edge states in chiral honeycomb nanoribbons. We show the evolution of the penetration depth during the change of the edge orientation from zigzag to armchair direction. The chiral nanoribbons are divided into two classes, zigzag-like and armchair-like ribbons. The penetration depth for armchair-like chiral ribbons is much larger than that for zigzag-like ones and can be used to estimate the finite-size induced gap. In order to achieve a nonlocal manipulation of the edge current by use of the edge magnetization, the intermediate chiral ribbon between the two classes is the best choice due to the coexistence of edge magnetization and considerable penetration depth. Our simulations show that the nonlocal transistor can be used as a the switch between the off-state and the on-state by the manipulation of the edge magnetization and can support a large edge current. The difficulty that the edge magnetization is unstable in the presence of a ballistic current is removed by this nonlocal manipulation of the edge channel.
